# On Croup

**Published:** 1869-04

**Authors:** Thomas Inman

**Affiliations:** Physician to the Liverpool Royal Infirmary


ON CROUP.—By THOMAS INMAN, M. D., Physician to the Liverpool Royal Infirmary, in *The Liverpool Medical and Surgical Reports, and Dublin Quarterly Journal of Medical Science, May.*

The following are the conclusions at which Dr. Inman arrives as to the treatment of this affection:—

"1. In slight cases no medicaments are necessary; hot, moist air and local warmth suffice; talking and laughing are to be deprecated so long as the laryngeal muscles are irritable; fever may be subdued by the free use of oil to the skin. 2. In more severe cases an emetic of ipecacuana will relax the mucous membrane, and thus put an end to that distressing dry stage with which we who suffer from catarrh are so familliar. 3. To reduce the irritability of the laryngeal muscles opiates may be used, both locally and generally. 4. We must next endeavor to remove, as far as possible, every irritant from the sensative spot; and to effect this, every breath which is inhaled should be of the temperature of the body, and moist as is the human breath. 5. Such symptoms as thirst and feverishness may be met by any drink the patient selects; it is certain that under such circumstances a child will neither select spirits, wine or ale. 6. The occasional inhalation of chloroform may be adopted, if the patient when first seen is in a very low condition."

